# Influenza A(H3N2) Subclade K During the 2025–26 Season and Differential Humoral Responses in Vaccinated Individuals Versus Vaccinated Convalescents in Israel

**DOI:** 10.3390/vaccines14100838

**Published:** 2026-09-23

**Authors:** Ital Nemet, Neta S. Zuckerman, Nofar Atari, Limor Kliker, Menucha Jurkowicz, Mayan Gilboa, Yael Weiss-Ottolenghi, Nathan Keller, Michal Stein, Gili Regev-Yochay, Yaniv Lustig, Michal Mandelboim

**Affiliations:** 1 The Central Virology Laboratory, Ministry of Health, Sheba Medical Center, Ramat Gan 52621, Israel; 2 Gray Faculty of Medical and Health Sciences, Tel Aviv University, Tel Aviv 6997801, Israel; 3 Sheba Pandemic Preparedness Research Institute, Sheba Medical Center, Tel Hashomer, Ramat Gan 52621, Israel; yael.ottolenghi@sheba.health.gov.il; 4 Infection Prevention and Control Unit, Sheba Medical Center, Tel Hashomer, Ramat Gan 52621, Israel; 5 School of Health Sciences, Ariel University, Ariel 4070000, Israel; 6 Pediatric Infectious Disease Unit, Sheba Medical Center, Ramat Gan 52621, Israel

**Keywords:** influenza, subclade K, vaccination, convalescents

## Abstract

**Background/Objectives:** During the 2025–26 influenza season, the antigenically distinct A(H3N2) subclade K emerged and spread globally, raising significant concerns regarding seasonal vaccine effectiveness. This study aimed to map antigenic and structural divergence of this novel strain compared to the 2025/26 A(H3N2) vaccine component. Furthermore, we investigated how this vaccine mismatch impacts functional humoral immunity by directly comparing antibody neutralization profiles between individuals with vaccine-induced protection and those with infection-acquired immunity. **Methods:** Phylogenetic and structural modeling analyses were performed on 2025–26 Israeli influenza samples to identify genetic distances and mutations in the hemagglutinin protein compared to the 2025/26 A(H3N2) vaccine component. Hemagglutination inhibition (HI) and microneutralization assays were conducted using 2025–26 winter serum samples from a cohort of vaccinated healthcare workers and vaccinated convalescents to quantify antibody responses against both vaccine-like and subclade K strains. **Results:** Phylogenetic analysis revealed a clear dominance of the subclade K strain in Israel during the winter of 2025–26. Significant vaccine mismatch was defined by eight newly identified hemagglutinin mutations in the circulating subclade K strains. Serological testing demonstrated that while vaccination alone produced a modest two-to-three-fold increase in antibody titers, baseline protection against subclade K remained critically low. Conversely, natural infection in vaccinated convalescents elicited a significantly stronger, broader immune response, demonstrating a 6-fold and 13-fold increase in neutralizing antibodies against the vaccine-like and subclade K strains, respectively. **Conclusions:** Natural infection provides a more pronounced increase in both binding and neutralizing antibodies compared to vaccination alone, which might contribute to broader cross-protection against antigenically drifted variants. These findings highlight the primary limitations of current influenza strain prediction and emphasize the urgent need for faster, more adaptable vaccine manufacturing platforms to better match actual circulating strains.

## 1. Introduction

The 2025 influenza season in the Southern Hemisphere exhibited a bi-phasic pattern. During the early winter months, H1N1pdm09 predominated, with fewer influenza B cases reported. However, in August 2025, Australia documented a pronounced emergence of a novel A(H3N2) variant, known as subclade K (formerly J.2.4.1). This epidemic curve underscores the dynamic interplay between viral competition, population immunity, and climatic drivers typical of austral winters [1,2]. Subclade K was first detected in a returning traveler in New York during June 2025 [3]. The early detection in international travel cohorts highlights the role of global mobility in seeding transmission chains and the importance of border testing programs as sentinel systems for variant discovery [4]. Rapid increases in tests positivity, outpatient influenza-like illness consultations, and hospital admissions were temporally coincident with the expansion of this lineage, suggesting a fitness advantage in the immunologic landscape [5]. Subclade K harbors distinct mutations in the hemagglutinin (HA) surface protein [5,6], constituting a significant antigenic drift which contributed to the mismatch of the A/Croatia/10136RV/2023, 2025/26 A(H3N2) vaccine component, and led to suboptimal viral inhibition by vaccinated human and ferrets’ sera [1,6,7,8]. In H3N2, antigenic drifts are driven primarily by amino acid substitutions within canonical antigenic sites (A–E) proximal to the receptor-binding domain of HA, as well as by the gain or loss of N-linked glycosylation motifs that modulate epitope accessibility [9]. Such changes can alter receptor-binding avidity, modify antigenic positions relative to vaccine strains, and reduce the breadth and potency of preexisting humoral immunity [10]. Subclade K quickly became the dominant strain across Australia and New Zealand, with indications of an unusually early widespread dissemination in the Northern Hemisphere [1,3,11,12]. In parallel, global genomic databanks recorded increasing subclade K submissions, enabling near-real-time phylogeographic tracking and risk assessment through the WHO GISRS framework [13]. The accelerated dissemination suggests sustained transmission in temperate climates during inter-seasonal periods, likely facilitated by travel networks and mass gatherings, and emphasizes the need for flexible strain selection timelines [14]. Influenza antigenic drifts that occur annually lead to compromised vaccine effectiveness and increased morbidity and mortality. Even modest antigenic divergence can translate into clinically meaningful reductions in vaccine effectiveness, particularly in older adults and young children, with downstream impacts on healthcare utilization and increasing winter mortality [14]. In Israel, subclade K was the dominant strain as well, six children suffered from this variant’s complications and died at the beginning of 2025–26 winter season. Here, we map subclade K in Israel compared to the 2025 vaccine-like strain using a phylogenetic analysis, highlighting subclade K’s predominance in Israeli samples during the 2025–26 winter, which demonstrates amino acid changes in the K subclade variant compared to a 2025 vaccine-like strain. Time-scaled phylodynamic analyses provides insight into local transmission dynamics, corroborating epidemiologic observations of rapid expansion [15]. Additionally, hemagglutination inhibition (HI) and microneutralization assays were performed to evaluate and compare antibody responses in vaccinated individuals and vaccinated convalescents. All sera samples are from the 2025–26 winter season and were assessed against both vaccine-like and subclade K strains. Sera were stratified by age, gender and time points around vaccination/infection. Fold-reduction analyses were visualized and quantified to determine the differences in the serologic landscape. This study aims to elaborate the implications of subclade K unique antigenic profile on vaccine effectiveness. By integrating genomic and immunologic datasets, we describe the mechanistic basis of reduced cross-protection and identify antigenic features. For this purpose, we propagated live seasonal viral isolates, which enable more accurate inference regarding real-world vaccine performance, and we utilized high-throughput serological assays. Our findings reveal intriguing differences in the responses to vaccination versus vaccination and infection and demonstrate the limitations of present influenza vaccines. In short, this research lay an evidentiary foundation for the development of next-generation rapid-response influenza vaccine platforms.

## 2. Materials and Methods

### 2.1. Study Population

The cohort includes healthcare workers in the Sheba Medical Center, named CHILL (Common Cold Healthcare Workers Immunological Longitudinal Learning). The study received approval from the institutional review board at the Sheba Medical Center (SMC-0196-23). An illustration and table with detailed information about the participants and procedures are found under Supplementary Figure S1A,B.

#### 2.1.1. Vaccinated Individuals

We collected serum samples from 7 individuals who received the Fluzone vaccine (Sanofi), before vaccination and approximately one month after vaccination.

#### 2.1.2. Vaccinated Convalescents

We chose 7 individuals who were vaccinated and infected at least one month following vaccination. Serum samples were obtained before vaccination and one month after recovery. RV Master Assay (Seegene, Seoul, Republic of Korea) was used for the detection of influenza in those participants, followed by a specific H1/H3 typing assay, which was performed as previously described [16]. The H3N2 strain was detected in all participants.

### 2.2. Sequencing and Bioinformatics Analyses

Influenza virus RNA from influenza-positive samples during the 2023–24 and 2025–26 winter seasons was amplified using whole-genome, influenza-specific primers [17]. Amplicons were used for library preparation with the Illumina DNA Prep kit (Illumina, San Diego, CA, USA). Library validation and mean fragment size were determined using the 4200 TapeStation via DNA HS D1000 kit (Agilent Technologies, Santa Clara, CA, USA). Sequencing was performed on the MiSeq i100 pro system (Illumina, San Diego, CA, USA).

Sequences were mapped to the influenza A vaccine strain (A/Croatia/10136RV/2023) using BWAmem, and consensus fasta sequences were generated with iVar [18], with positions supported by sequencing depth < 5 masked as Ns.

Phylogenetic analysis was performed using the hemagglutinin (HA) genes from each sample.

The tree was constructed using the Nextstrain Augur 34.1.4 pipeline under a generalized time-reversible substitution model and visualized with Auspice [19].

### 2.3. Protein Illustration

The HA protein structure was visualized using genius-prime software version 2026.1.3 (Schrodinger, New York, NY, USA). Influenza sequences were retrieved from the EpiFluTM database of the GISAID.

### 2.4. Hemagglutination (HI) Assays

Serology samples were treated with the RDE treatment protocol (DENKA, Chuo-ku, Tokyo, Japan) followed by heat inactivation at 56 °C for 30 min, and the elimination of unspecific sera-red blood cells interactions. Thereafter, sera were tested at an initial dilution of 1:20, and up to a final dilution of 1:2560. Human red blood cells (O+) were used. (Same donor for all experiments). Influenza strains that were used are as follows: 2025/26 H3N2 NH vaccine component and subclade K strain (A/Israel/619/2024 and A/Israel/17832/2025, respectively). The viral antigens preparation was performed by incubation of 1 mL of each live isolate with 10 microliter formaldehyde 1% for 1 h at 37 °C. Standardized viral antigen was freshly prepared on the days the assay was performed to a standard dilution of 4 HA units. The HI assay was performed as previously described [20]. For the geometric mean titer (GMT) calculation, any samples with a negative result by HI assay were assigned a base titer of 1:10.

### 2.5. Microneutralization Assays

Neutralizing antibody titers against the 2025/26 H3N2 NH vaccine component (A/Israel/619/2024) and subclade K strain (A/Israel/17832/2025) were measured in the healthcare workers sera. The two influenza isolates were propagated on MDCK-CIAT cells with DMEM medium added with 2% fetal calf serum and trypsin from porcine pancreas (3.33 µg/mL) (Sigma, St. Louis, MI, USA). Neutralizing activity against the two influenza isolates was assessed using a Microneutralization Assay. MDCK-CIAT cells (WHO) (25 × 10^3^ cells/well) were seeded in sterile 96-well plates containing DMEM medium added with 10% fetal calf serum and incubated at 37 °C for 24 h. Subsequently, 100 TCID_50_ of each strain was incubated with heat-inactivated (56 °C for 30 min), serially diluted patient sera for 60 min at 37 °C. The virus–serum mixtures were then added to the MDCK-CIAT cells. Cells with virus only served as positive control, cells only as negative control. Plates were incubated at 37 °C for five days, and neutralizing capacity was determined as the highest serum dilution at which no cytopathic effect was observed [21].

### 2.6. Statistical Analysis

GMT with 95% confidence intervals (CI) was used to assess the means of antibody titers reported for each group for each isolate. Differences between paired pre- and post-exposure antibody titers were assessed using the Wilcoxon signed-rank test (Figure 3, within-group analysis). Fold change was assessed as the geometric mean ratio (GMR), calculated from log-transformed post/pre titers and back-transformed to the original scale, with 95% CI.

Differences in the log-transformed fold change between vaccinated and infected participants were assessed using the Wilcoxon rank-sum test and subsequent pairwise analyses (Figure 4, between-group analysis). All statistical analyses were performed and Graphics were created with GraphPad Prism 9.5.1 and R Statistical Software (version 4.5.2; R Foundation for Statistical Computing, Vienna, Austria).

## 3. Results

### 3.1. Phylogenetic Analysis of Israeli Samples, Winter 2025–26

Phylogenetic analysis was conducted using A(H3N2)-positive samples from hospitalized and ambulatory patients in Israel during the 2025–26 winter season and the 2023–24 winter season (Figure 1). This analysis demonstrated clear genetic distances between the two subclades, marked by eight characterized HA mutations in subclade K strains.

According to the structural model of the hemagglutinin, (Figure 2), circulating 2023–24 Israeli strains (subclade J, (A/Israel/619/2024 as reference)) closely resemble the 2025/26 vaccine strain (A/Croatia/10136RV/2023) with only two antigenic changes (N145S and L409I). Thus, A/Israel/619/2024 was referred as the vaccine-like strain. Importantly, the N145S mutation in the vaccine-like strain was detected among all Israeli samples of winter 2023–24 that were sequenced and are illustrated in the phylogenetic tree. In contrast, subclade K strains (A/Israel/17832/2025 as reference) diverged significantly, with numerous mutations highlighted in different colors, indicating a limited antigenic match between the 2025/26 vaccine A(H3N2) component and the 2025 circulating strain.

### 3.2. Humoral Responses and Viral Inhibition in Vaccinated Individuals and Vaccinated Convalescents During Winter Season of 2025–26

Healthcare workers from Sheba Medical Center were selected as the study cohort, as described in Supplementary Figure S1B. Participants were divided into two groups:

Seven vaccinated individuals, (Figure 3A, before and after vaccination) and seven vaccinated individuals who also contracted influenza (Figure 3B, before vaccination and after infection). Antibody responses were quantified against a previously propagated 2025–26 vaccine-like strain and a subclade K strain using HI and microneutralization assays (Methods and cohort description in Supplementary information).

Vaccinated individuals demonstrated a statistically significant ~2-fold increase in post-vaccination hemagglutinin inhibition for both strains evaluated (*p* = 0.03). Microneutralization assays exhibited significant suppression of viral infectivity, with a ~3-fold increase following vaccination against each strain (*p* = 0.03). Importantly, markedly higher titers against the vaccine-like strain were detected in both assays, before and after vaccination, compared to subclade K’s titers which remained low even after vaccination. (*p* = 0.02). Prior to vaccination, neutralizing antibodies against the vaccine-like strain were significantly higher for the vaccinated group, compared to the vaccinated convalescent group (*p* = 0.016). Vaccinated convalescents showed significant hemagglutinin inhibition with a ~4-fold increase for the two strains (*p* = 0.03). Microneutralization assay results illustrate noticeably higher neutralizing antibody titers for vaccinated convalescents, with a ~6- and 13-fold increase for the vaccine-like and subclade K strains, respectively (*p* = 0.02).

Figure 4 depicts the fold changes in HI and the neutralizing antibody response between vaccinated individuals and vaccinated convalescents against each strain, underscoring a significant elevation in neutralizing antibodies against subclade K among convalescent individuals. (*p* = 0.01).

## 4. Discussion

The 2025–26 influenza season in the northern hemisphere has been marked by the early and rapid proliferation of the A(H3N2) subclade K, which has exhibited heightened activity across multiple countries, effectively suppressing other circulating A(H3N2) strains [1,3,8,11,12,22]. This pattern suggests a transmission advantage conferred by this new subclade’s antigenic profile. Phylodynamic analyses in analogous settings have shown that H3N2 lineages frequently achieve dominance within weeks under permissive conditions of travel-mediated seeding and heterogeneous population immunity, thereby outcompeting co-circulating clades with narrower antigenic breadth [23]. Compared to other seasonal influenza strains, influenza A(H3N2) demonstrates the highest evolutionary rate and is associated with more severe epidemics, with increased morbidity and mortality, particularly among older adults [24]. The elevated substitution rate in H3N2—concentrated in the hemagglutinin (HA) head—accelerates antigenic drift relative to H1N1pdm09 and influenza B, producing frequent antigenic shifts that degrade cross-protective immunity accrued from prior infections and vaccinations. Mechanistically, such an advantage likely reflects a constellation of HA substitutions that simultaneously diminish binding by preexisting antibodies and modulate receptor-binding avidity, thereby improving viral fitness, while a cost in replication or transmissibility is not necessarily found [9,10,25].

Recurrent findings show that egg-based H3N2 components demonstrate a reduced antigenic match compared with cell-based or recombinant counterparts, due to egg-adaptive mutations. These egg-acquired changes increase antigenic distance between vaccine and wild-type viruses in HI/neutralization assays, contributing to lower vaccine effectiveness when H3N2 predominates [26,27]. Even without a large “cluster jump,” the combination of incremental drift within 3C.2a1b and egg-adaptation can yield clinically relevant mismatch in specific locales/seasons. Comparative effectiveness studies suggest that cell-culture and recombinant HA vaccines partially mitigate this mismatch by preserving human-adapted glycosylation and receptor-binding properties, though gains vary by season and age group. Programs that use cell-based or recombinant H3 components may mitigate part of this gap [28,29].

In this study, we focused on antigenic characteristics and humoral responses against two seasonal influenza variants, one from the 2023–24 winter season (A/Israel/619/2024), and the other from the 2025–26 winter season (subclade K). Selecting concurrent clinical isolates rather than vaccine seed strains provides a more accurate reflection of the vaccines’ actual effectiveness and the related annual population morbidity, as these isolates retain the glycosylation patterns and receptor-binding properties of circulating viruses [30,31].

As demonstrated in the structural analyses (Figure 2) and previously described by Zambon et al. [3], subclade K harbors numerous amino acid substitutions in the hemagglutinin protein relative to the current vaccine strain. Several of these mutations (T135K/S144N/N158D/I160K/T328A and K2N/Q173R/S378N) are clustered within canonical antigenic sites A and B near the receptor-binding domain, consistent with known patterns of H3N2 antigenic drift that prioritize escape at epitopes most commonly targeted by human neutralizing antibodies [9,25,32].

T135K disrupts the N-linked glycosylation sequon (N-X-S/T) at HA1 133–135, removing the N133 glycan, which exposes antigenic site A and can increase receptor-binding avidity. Also, in 3C.2a1/3C.2a1b H3N2 viruses, substitutions at HA1 160 (I/T160K/K160T) toggle the N158 glycan at site B. This toggling reshapes the steric shielding of site B, alters antigenic cartography positioning, and affects the balance between antibody escape and receptor binding. Together, glycan loss at 133 and toggling at 158 reconfigure shielding of HA-head epitopes—exposing site A and variably shielding site B—thereby shifting antigenicity and receptor avidity [33,34,35].

These data, coupled with our phylogenetic analysis and observed reductions in viral inhibition by vaccinated ferret sera [1,6,8,12], raise concerns regarding partial immune escape and a consequent reduction in vaccine effectiveness. Ferret antisera, while not perfectly concordant with human immunity, frequently anticipate antigenic relationships observed in human post-vaccination sera and remain a core element of global influenza vaccine strain selection workflows [30,36].

Children under nine years of age usually receive two vaccine doses upon their first influenza vaccination, because previously unexposed or unvaccinated individuals often demonstrate a suboptimal adaptive immune response. This past winter, six Israeli children under the age of ten died following infection with the subclade K influenza variant, most likely during their first encounter with the virus. Notably, only one of these children had been vaccinated during the current season. Age-specific susceptibility in pediatric cohorts is further shaped by immune imprinting from first influenza exposures; when the circulating strain’s HA diverges substantially from the imprinting subtype, the risk of severe outcomes may increase [37]. These pediatric deaths highlight the virulent potential of subclade K or other novel variants with multiple mutations and are consistent with heightened susceptibility in immunologically naïve cohorts. In addition to better vaccination strategies, rapid identification of antigenic drift through integrated genomic and serologic surveillance is essential for guiding clinical preparedness and public health messaging [4,30].

HI and microneutralization assays were conducted to compare the humoral immune response elicited by vaccination with that induced by natural infection, against both subclade K and a 2024 seasonal circulating strain, which is of high antigenic resemblance to the 2025/26 A(H3N2) vaccine component.

While HI is an established, rapid, and straightforward assay for detecting binding antibodies, microneutralization is more sensitive and biologically relevant, measuring broad-ranged functional antibodies that actively prevent infection. HI predominantly measures antibodies that block HA binding to sialylated receptors on RBCs, whereas neutralization integrates multiple mechanisms—including the inhibition of attachment and fusion, interference at the HA–NA interface, and contributions from stalk-directed antibodies—thereby providing a more comprehensive assessment of protective capacity [38].

Our findings revealed that vaccinated individuals exhibited a modest, 2- to 3-fold increase in antibody response against both subclade K and the 2025 vaccine-like strain. Interestingly, prior to vaccination, high neutralizing antibody levels against the vaccine-like strain were observed. Vaccinated participants in our cohort were likely to have received routine influenza vaccinations in previous years, as healthcare workers in our hospital. The high neutralizing antibody levels observed most probably reflect previous exposures and pre-existing immunity among frequent vaccine recipients. Repeated annual vaccination can shape antibody landscapes toward conserved or recurrent epitopes, yielding sustained baseline neutralization against antigenically proximate strains [39,40]. However, baseline titers against the novel subclade K were five times lower compared to those against the vaccine-like strain. Therefore, post-vaccination levels are still relatively low and might not be protective. Although vaccination increased antibody titers against both subclade K and the vaccine-like strain, the reduced baseline titers against subclade K suggest that while vaccine-elicited antibodies provide some degree of protection against influenza A viruses [10], their efficacy may vary depending on the antigenic similarity between the vaccine and newly emerging viral strains.

Here we show that vaccinated convalescent individuals demonstrate a more significant elevation in both HI and neutralizing antibodies against both viral isolates, with a stronger pattern in the microneutralization assays. This is particularly reflected by significantly higher antibody titers against subclade K found among vaccinated convalescent individuals, compared with the vaccinated group. The augmented effect in vaccinated convalescents likely reflects hybrid immunity, wherein infection reactivates and diversifies memory B cell repertoires established by vaccination, promoting broader recognition across HA head and stalk epitopes [39,40]. High neutralizing antibody titers against the vaccine-like strain were observed in vaccinated individuals, as they express higher baseline levels prior vaccination. However, the fold change is remarkably higher for convalescents. These patterns are consistent with antibody landscape studies showing that infection events often induce broad “back-boosting” of responses to previously encountered antigens, while sharpening responses to the infecting strain at the same time [39]. All vaccinated individuals who eventually contracted influenza were detected with ~6-fold lower initial antibody titers against the vaccine-like strain. This finding indicates a cross-protection when a sufficient number of pre-existing antibodies is present. While observational, this threshold-like phenomenon mirrors established correlations between pre-season titers and reduced infection risk, supporting the utility of sero-epidemiologic monitoring in high-risk groups [41,42].

According to Figure 3, while the neutralization assay revealed robust differences in antibody titers levels between research groups before and after vaccination, HI results demonstrate a modest effect, to a much lesser extent. HI probes only the blockade of HA attachment to RBC sialic acids and is sensitive to non-antigenic factors (HA receptor avidity, RBC species, and neuraminidase-mediated binding); contemporary H3N2 often weakly or aberrantly agglutinates RBCs, compressing HI titers and masking antigenic differences. By contrast, cell-based neutralization measures prevention of infection and integrates multiple mechanisms (attachment and fusion blockade, interference with HA–NA, stalk-directed responses), with fewer RBC artifacts and a broader dynamic range, thus better resolving mismatches and yielding larger fold-differences [43,44,45,46,47]. Consequently, neutralization assays are increasingly favored for fine-resolution antigenic characterization of drifted H3N2 lineages, especially when guiding vaccine strain updates [25].

Overall, the initial titers against subclade K were low among both research groups, due to the major antigenic changes in this new variant. However, a dramatic increase in antibody levels was observed in vaccinated convalescent individuals, compared with the vaccinated group, which demonstrated modest elevation.

Importantly, while vaccination might result in higher antibody titers against past variants, influenza infection induces robust antibody production against current, circulating strains (subclade K), accompanied by the reactivation of humoral response against older strains (vaccine-like strain). Those differences can be explained by the fact that infection induces a massive, broad-spectrum antibody response targeting diverse viral epitopes, as opposed to a strain-specific immunity generated by conventional vaccines.

Nevertheless, despite those notable antigenic shifts, recent reports suggest that vaccine effectiveness this winter, as reflected by outcomes such as emergency department visits and hospital admissions, remained within typical ranges for all age groups [8,48,49,50,51].

Presenting small cohorts, this study has a main limitation that should be considered when interpreting the findings. The sample size constrains statistical power and limits stratified analyses by age, comorbidity, and prior exposure. Moreover, cellular immune response (including CD4+/CD8+ cells) is a crucial factor involved in protection from disease severity, and it was not addressed in this study. Robust T cell responses targeting internal proteins (NP, M1) can mitigate disease severity independent of humoral titers, particularly in the face of antigenic drift [52], and should be incorporated in future prospective designs. These drawbacks warrant further investigation in prospective studies with larger cohorts.

## 5. Conclusions

Collectively, our preliminary data indicate that natural infection confers a more pronounced increase in both binding and neutralizing antibodies than vaccination, which may contribute to broader protection against antigenically separate variants. While higher antibody levels are expected in individuals who were vaccinated and infected, this study is unique and valuable, since it compares humoral responses between two seasonal influenza variants, using two serological approaches.

Our results highlight the main limitations of influenza vaccines—the unsuccessful prediction of future circulating strains, and frequent mismatches between the vaccine strains and actual circulating seasonal strains. These drawbacks can result in high morbidity and mortality when more virulent, antigenically distinct influenza variants emerge. Thus, there is an urgent need for a more adequate, faster vaccine preparation process. Future vaccine strategies may need to incorporate conserved epitope-focused immunogens and platforms that minimize egg-adaptive artifacts, such as adaptable mRNA and recombinant protein systems capable of quick antigen updates, which are based mainly on the strains circulating during winter season in the opposing hemisphere [53,54].

Nonetheless, our results suggest that the 2025/26 A(H3N2) vaccine component shares some epitopes with the subclade K strain. Accordingly, annual influenza vaccination continues to be strongly recommended.

## Figures and Tables

**Figure 1:**
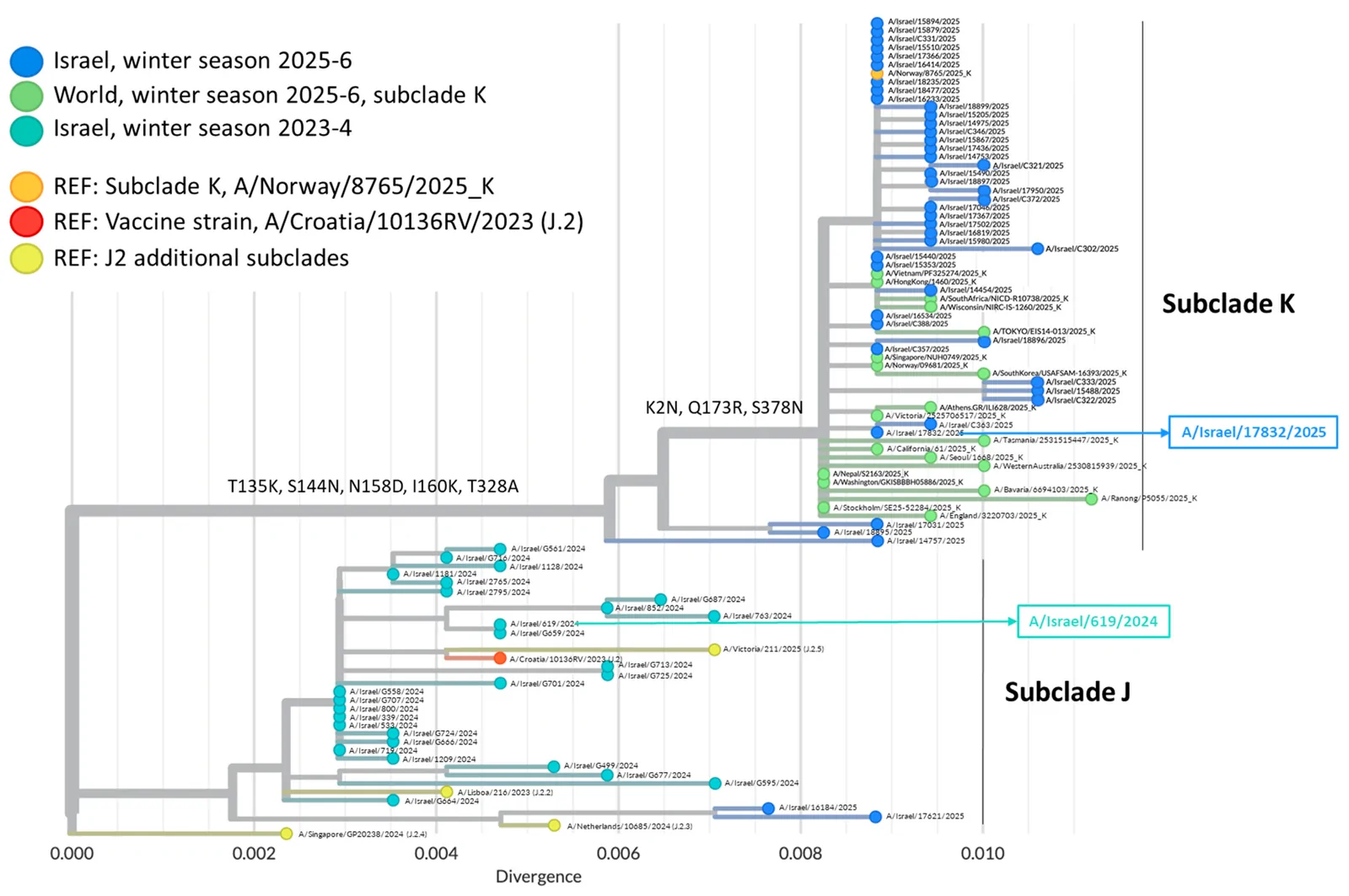
Phylogenetic analysis of seasonal influenza strains. Phylogenetic divergence tree of the HA gene including Israeli influenza sequences from the 2025–2026 (n = 44) and 2023–2024 (n = 27) seasons, alongside reference sequences representing the vaccine strain administered during these seasons (A/Croatia/10136RV/2023) and subclade K (A/Norway/8765/2025_K). All sequence results are published in GSAID. Amino acid substitutions defining subclade K are indicated. Tip labels include the strain name, country of origin, and year of sample collection (where available).

**Figure 2:**
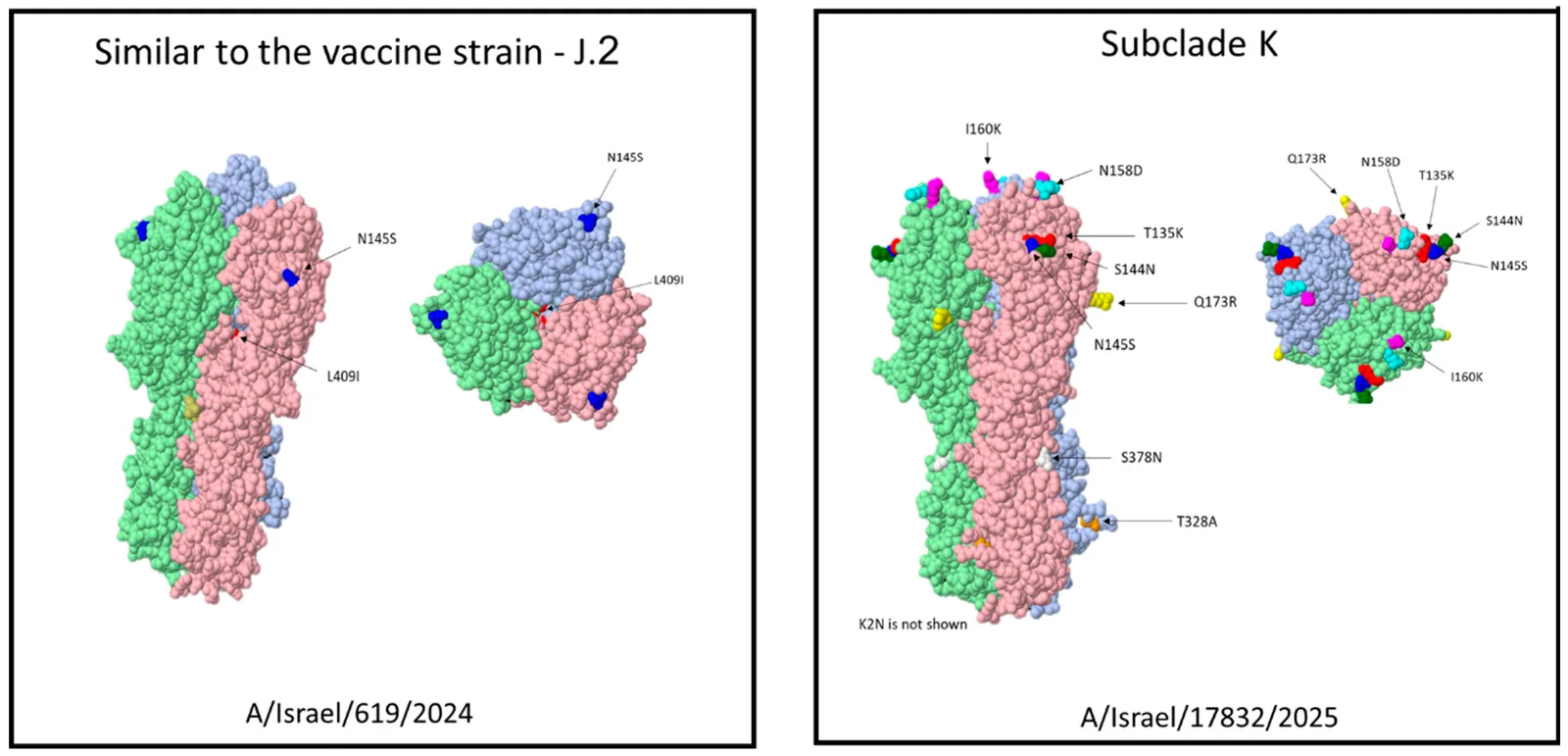
Structural models of hemagglutinin amino acid substitutions. The 2025/26 A(H3N2) NH vaccine-like component (A/Israel/619/2024, (**left**)) and subclade K strain (A/Israel/17832/2025, (**right**)) were compared to the vaccine strain (A/Croatia/10136RV/2023). Hemagglutinin three subunits are depicted in light-blue, green and pink. All amino acid substitutions are highlighted in different colors.

**Figure 3:**
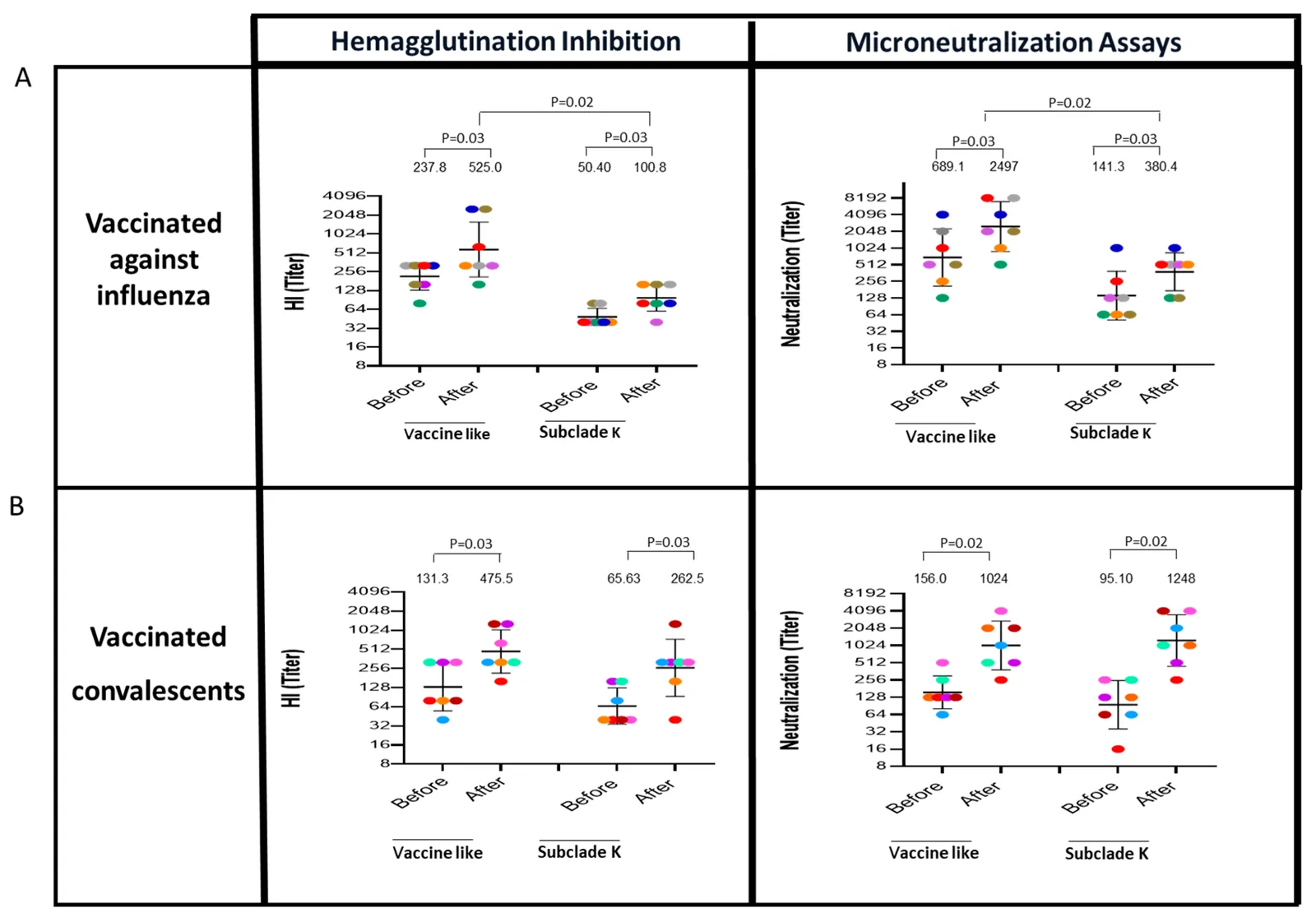
Comparison between antibody titers against subclade K strain and a vaccine-like strain in vaccinated individuals and vaccinated convalescents during the 2025 Israeli winter season. Cohort is presented in Supplementary Figure S1B. (**A**) Influenza antibody titers against vaccine-like strain and subclade K strain, before and after vaccination as measured via hemagglutination inhibition (**left**) and microneutralization (**right**). (**B**) Neutralizing antibody titers against vaccine-like strain and subclade K strain, before vaccination and after infection as measured via hemagglutination inhibition (**left**) and microneutralization (**right**). Each dot is a different color, representing each participant.

**Figure 4:**
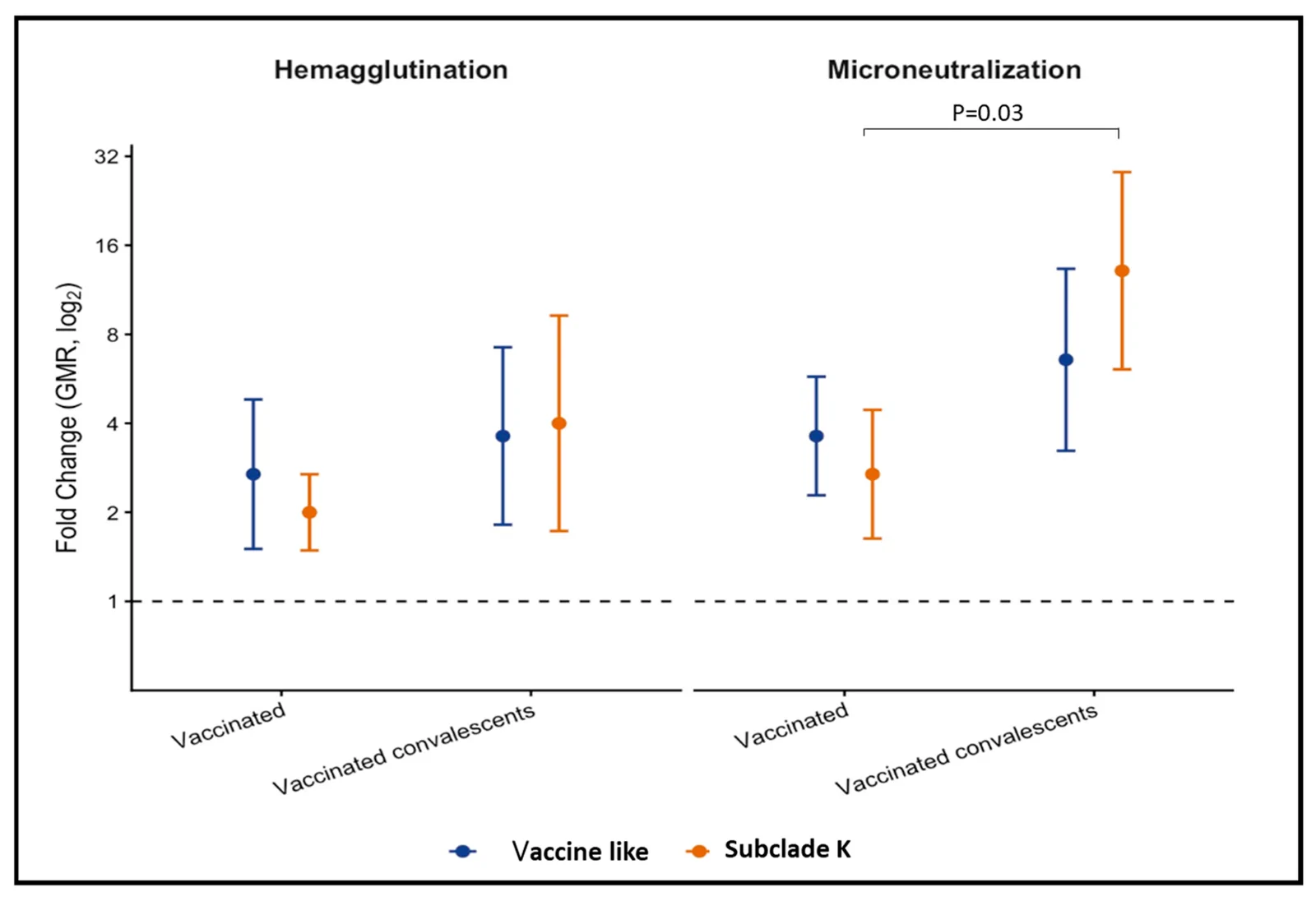
The fold change in antibody titers against subclade K strain and a vaccine-like strain, in vaccinated individuals and vaccinated convalescents. Statistical significance of hemagglutination inhibition titers against each strain among the two research groups ((**left**), not statistically significant). The statistical significance of influenza neutralizing antibody titers against each strain among the two research groups (**right**). The reference line at fold change 1 indicates no change between pre- and post- exposure antibody titers.

## Data Availability

The original contributions presented in this study are included in the article/Supplementary Material. Further inquiries can be directed to the corresponding author.

## Supplementary Materials

The following supporting information can be downloaded at: https://www.mdpi.com/article/10.3390/vaccines14100838/s1, Figure S1. (**A**) The illustration describes blood sampling timelines around vaccination and infection, in vaccinated individuals and vaccinated convalescents; (**B**) characteristics of cohorts by age and gender, and antibody titers from before vaccination and after vaccination or infection, for vaccinated individuals and vaccinated convalescents, respectively.
