# Supplementary material for: Influenza A(H3N2) Subclade K During the 2025–26 Season and Differential Humoral Responses in Vaccinated Individuals Versus Vaccinated Convalescents in Israel
Source: Vaccines (Basel). 2026 Sep 23;14(10):838. doi: 10.3390/vaccines14100838 (PMC13709554; doi:10.3390/vaccines14100838)
Supplement: Supplementary file 1 [file vaccines-14-00838-s001.zip › vaccines-4523688-supplementary.pdf]

Supplementary material, figure S

A

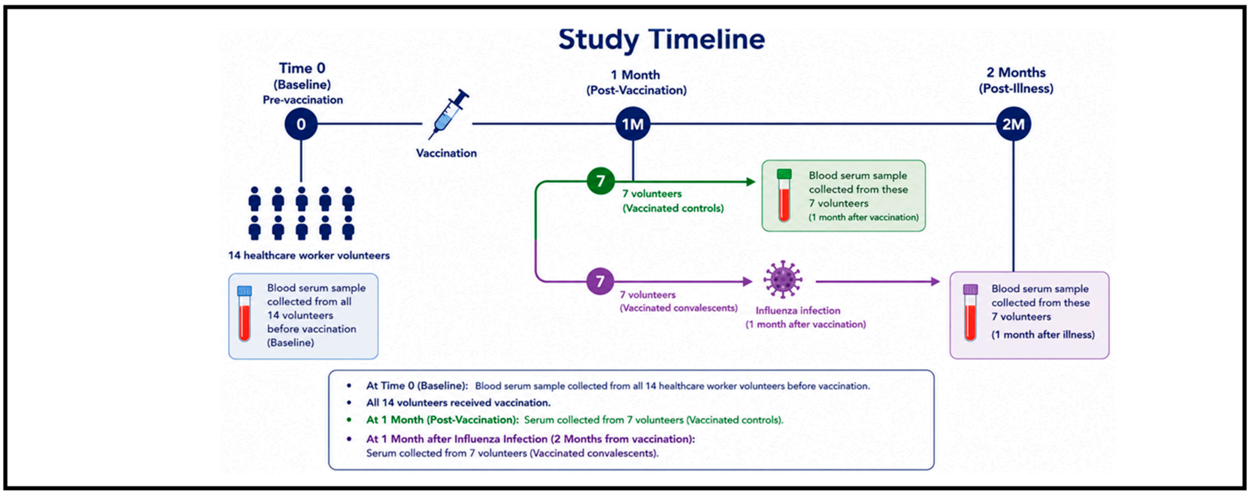

B

							Hemagglutination Inhibition				Microneutralization Assays			
	Number	Color	Age	Gender	Days illness after vaccine	Days after vaccine	J-2		Sabclase K		J-2		Sabclase K	
Vaccinated against influenza	1		52	Female	X	29	320	640	40	80	1024	8192	256	512
	2		60	Female	X	31	160	320	40	40	512	2048	128	512
	3		48	Female	X	77	80	160	40	80	128	512	64	128
	4		25	Female	X	69	320	2560	40	80	4096	4096	1024	1024
	5		32	Female	X	77	160	320	40	160	256	1024	64	512
	6		40	Female	X	69	320	320	80	160	2048	8192	128	512
	7		24	Female	X	74	320	2560	80	160	512	2048	64	128
Vaccinated convalescents	1		68	Male	44	85	80	160	40	40	128	256	16	256
	2		41	Female	18	37	320	320	160	320	256	512	256	1024
	3		31	Female	20	30	40	320	80	320	64	1024	64	2048
	4		58	Female	49	83	80	320	40	160	128	2048	128	1024
	5		61	Female	31	70	320	1280	160	320	128	512	128	512
	6		52	Female	29	84	80	1280	40	1280	128	2048	64	4096
	7		70	Female	45	85	320	640	40	320	512	4096	256	4096

Figure S1A: The illustration describes blood sampling timelines around vaccination and infection, in vaccinated individuals and vaccinated convalescents.

Figure S1B: Characteristics of cohorts by age and gender, and antibody titers from before vaccination and after vaccination or infection, for vaccinated individuals and vaccinated convalescents, respectively.
